# Audit and feedback in community pharmacy: An untapped strategy of quality improvement

**DOI:** 10.1177/17151635211056528

**Published:** 2021-11-05

**Authors:** Charlotte G. Boone, Andrea C. Bishop, Noah Ivers, Laura Desveaux, Mina Tadrous

**Affiliations:** Centre for Addiction and Mental Health; Toronto, Ontario; the Nova Scotia College of Pharmacists, Halifax, Nova Scotia; Women’s College Hospital Research Institute, Toronto, Ontario; Women’s College Hospital Research Institute, Toronto, Ontario; Trillium Health Partners, Mississauga, Ontario; Women’s College Hospital Research Institute, Toronto, Ontario; University of Toronto, Toronto, Ontario

Community pharmacists have a significant impact on the safe and effective use of medication through assessing the appropriateness of all aspects of a prescription. Despite improved transitions of care, management of chronic diseases and medication adherence from pharmacist-led interventions,^1-3^ the day-to-day clinical performance of pharmacists is not being measured. In a role that has been described as working within a “silo” of the larger health care system,^4^ it is challenging for community pharmacists to benchmark their professional performance and identify areas for improvement. Community pharmacists remain an untapped resource when it comes to accelerating health system improvement, with little if any attention paid to supporting pharmacists in optimizing the quality of care provided in their practice. Despite the availability and opportunity to use routinely collected, accessible data to give community pharmacists the tools to exercise quality improvement in their practice, there are currently no standardized audit and feedback (A&F) mechanisms.

## What is audit and feedback?

A&F involves measuring a practitioner’s performance in selected areas and comparing it to professional standards or targets. A&F is nonpunitive and seeks to improve clinical practice. With roots in behavioural science, A&F is an evidence-based method used across health care settings. The general premise of A&F is that when a health care practitioner is provided with a report of their behaviour in comparison to best practice and their peers, they will understand in which area(s) there is opportunity to improve and be motivated to change their behaviour.

A systematic review and meta-analysis of A&F on professional practice and health care outcomes found that A&F generally leads to small (a 4.3% increase in compliance with desired practice) but potentially important improvements in professional practice.^5^ It was also found that A&F is most effective when baseline performance is low, the source is a supervisor or colleague, it is provided more than once, it is delivered in both verbal and written formats and it includes both explicit targets and an action plan. In Quebec, a randomized trial assessed the impact of A&F for community pharmacists. This trial found that pharmacists receiving asthma feedback as a one-time written report had 1.6 times the chance of billing for services recommending changes to patients’ medication.^6^

There is a breadth of evidence of the efficacy for A&F interventions for other health care professions. In a study from the United Kingdom, pharmacists were leveraged as feedback providers and communicated with general practitioners regarding the appropriateness of their prescribing (PINCER trial). This approach led to improved physician practices in all 3 clinical criteria measured.^7^ Closer to home, Ontario physicians can access their metrics through an online dashboard, MyPractice Ontario, to review their performance in relation to their colleagues in several areas, including routine screening rates and opioid prescribing. In an attempt to reduce harm caused by the overuse of antipsychotic medications in nursing homes, an Ontario study found that accessing MyPractice feedback reports significantly reduced physicians’ antipsychotic prescribing.^8^

These interventions have also been shown to help reduce costs to health care systems. For example, the return on investment from the MyPractice report in Ontario long-term care was estimated to be $1.22 for every $1 spent.^9^ This is unsurprising, considering system-wide A&F interventions are cheaper than other quality improvement interventions, primarily because they leverage routinely collected administrative data to develop reports.

The efficacy of A&F strategies suggests that the establishment of an A&F program targeted to community pharmacists might prove valuable. Opioids represent an established example of the potential improvement in patient outcomes from pharmacist interventions^10-12^ and is consistently selected as one of the quality indicators by the Ontario College of Pharmacists.^13^ The actionability in this space allows for quality improvement by pharmacists that has the ability to profoundly affect this public health crisis. Pharmacists are perfectly positioned to flag inappropriately high starting doses, suggest reduced quantities, dispense naloxone kits, communicate risks to patients and ensure concomitant therapy is appropriate, all of which have been shown to improve patient outcomes.^10-12,14^ With all Canadian jurisdictions capturing electronic claims submissions, the data required to formulate an audit for community pharmacists could be efficiently consolidated.

## What is currently happening?

There are few A&F initiatives globally that target community pharmacies. The Pharmacy Quality Alliance (PQA) is a nonprofit organization in the United States that investigates quality improvement indicators to report adherence, appropriate medication use, medication safety and medication therapy management measures.^15^ The PQA recently partnered with Green Shield Canada to offer a “Value-Based Pharmacy” program that provided an A&F scorecard to pharmacies. Although the A&F aspect was generally well received, the performance measures being tied to pharmacy reimbursement have been criticized.^16^

While there are no widespread A&F policy-level initiatives in Canada, an A&F pilot project for community pharmacies in Ontario was jointly launched by the Ontario College of Pharmacists and Health Quality Ontario in 2019 to measure 3 evidence-based audit indicators. The first indicator is the annual proportion of unique individuals newly initiated on opioids at a daily dose above 50 mg morphine equivalents (MMEs), a threshold associated with increased long-term use and opioid-related harms.^14,17-21^ The second indicator is hospital visits for opioid poisonings among patients who are actively treated with an opioid prescription, an area where pharmacists may intervene if unsafe doses or problematic use is suspected.^22-24^ Finally, the third indicator is the proportion of patients with a claim for a medication review (MedsCheck) presenting to a community pharmacy within 7 days of discharge from hospital.^25^ These indicators are measured using provincially held billing and hospital data. Presently, data are only available on an aggregate provincial and regional level. In contrast to the Value-Based Pharmacy program, these measures are not tied to reimbursement. Pharmacies are thus able to contextualize the indicators in relation to their business model and patient population, using the reports solely to improve patient outcomes. To date, the results of this pilot project have demonstrated community pharmacists’ tendency to overestimate their performance on quality indicators: self-reported estimates were lower for opioid prescribing (12%) and much higher for medication reviews (33%) than the calculated provincial values. The pilot project highlights the feasibility of using routinely collected health data to measure these indicators and suggests a disconnect between perceived and actual performance across several quality indicators. The findings from this pilot will be used to scale the A&F program within Ontario, with further learnings being shared with other jurisdictions in Canada in the hopes of scaling nationally.

## How can the system leverage A&F to help improve pharmacy practice?

As their professional role continues to expand from primarily dispensing to the provision of clinical services, pharmacists will require the knowledge and tools to provide safe, quality care. At this turning point, A&F will lead to direct benefits to patient care and holds the potential to create a ripple effect by positively affecting the culture of pharmacy practice to embody continuous quality improvement.

Professional audit and feedback mechanisms are long overdue in community pharmacy practice. Billing data are becoming more easily retrievable across Canada and should be leveraged to build a culture of continuous quality improvement. This can benefit the wider health care system and population by fostering improved practices. Canadians deserve to have access to pharmacy professionals who have the resources and support to identify where they can improve in their practice. ■
